# Simultaneous Gastric and Duodenal Erosions due to Adjustable Gastric Banding for Morbid Obesity

**DOI:** 10.1155/2014/146980

**Published:** 2014-05-05

**Authors:** Dimitrios K. Manatakis, Ioannis Terzis, Ioannis D. Kyriazanos, Ioannis D. Dontas, Christos N. Stoidis, Nikolaos Stamos, Demetrios Davides

**Affiliations:** 1st Surgical Department, Athens Naval and Veterans Hospital, 70 Deinokratous Street, 11521 Athens, Greece

## Abstract

Erosion is an uncommon but feared late complication of adjustable gastric banding for morbid obesity. A high index of clinical suspicion is required, since symptoms are usually vague and nonspecific. Diagnosis is confirmed on upper gastrointestinal endoscopy and band removal is the mainstay of treatment, with band revision or conversion to other bariatric modalities at a later stage. Duodenal erosion is a much rarer complication, caused by the connection tubing of the band. We present our experience with a case of simultaneous gastric and duodenal erosions, managed by laparoscopic explantation of the band, primary suture repair of the duodenum, and omentopexy.

## 1. Introduction

Laparoscopic adjustable gastric banding (LAGB) is a well-established restrictive procedure, still popular among many bariatric surgeons, because of its adjustability, reversibility, and preservation of gastrointestinal tract continuity [1–4]. While perioperative complications are minimal, compared to other bariatric modalities, it has a relatively high reoperation rate. Recent reviews, studying long-term results and complications, reveal a failure rate between 10 and 20% in the short run and 40% in the long run and an incidence of 12–48% of device-related complications [3]. These include early (band obstruction, gastric perforation, wound infection, and bleeding) and late (band slippage, pouch enlargement, port/tubing complications, and gastric erosion) complications, leading generally to unacceptable weight loss rates and requiring revision or conversion to other modalities [3].

With a reported incidence of 1–3%, gastric erosion is a relatively rare but potentially life-threatening complication [3–5]. We present our experience with a case of simultaneous gastric and duodenal erosions, caused by the band and the connection tubing, respectively.

## 2. Case Presentation

A 34-year-old female Caucasian patient presented at the emergency department with a 5-day history of protracted vomiting and epigastric pain. She had undergone LAGB (Bioring, Cousin Biotech, France) for morbid obesity 4 years before (height: 165 cm, weight: 100 kg, BMI: 36.7, and comorbidities: arterial hypertension, dyslipidemia, and low back pain) resulting in a weight loss of 30 kg (85% EBWL). The original band had been replaced laparoscopically 3 years after initial surgery, due to connection tubing failure.

**Figure 1:**
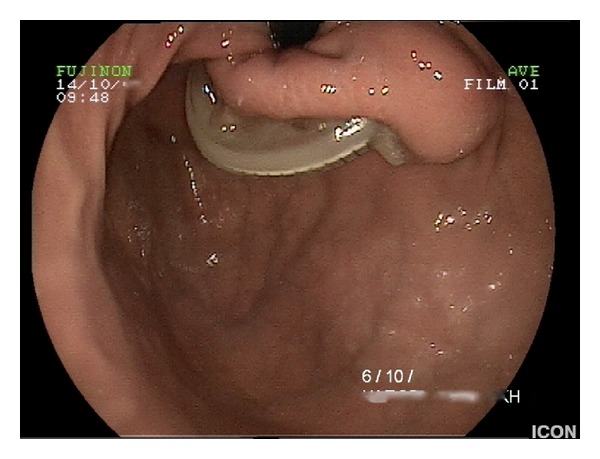
Retroflexed inspection of the gastric fundus and gastric erosion by the band.

**Figure 2:**
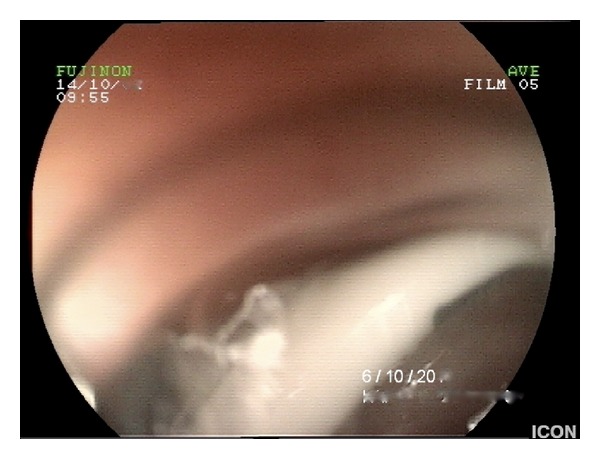
Duodenal erosion by the connection tubing.

Clinical examination revealed tachycardia, mild tenderness over the epigastrium, and signs of dehydration. Laboratory tests and plain abdominal radiographs were within normal range. Abdominal ultrasonography and CT scans were inconclusive; however the band was visible on upper GI endoscopy (Figure 1), protruding partially into the gastric lumen (stage 2 according to Nocca classification, >50% of the band free in the gastric lumen [6]). Endoscopy of the duodenum revealed concurrent erosion of the first part by the connection tubing (Figure 2).

The patient consented to surgical treatment and removal of the band. On laparoscopy, adhesions were taken down and the band was dissected free, cut near the buckle, and extracted. The duodenal erosion, about 1 cm in length, was repaired primarily by interrupted, absorbable polyglactin (Vicryl, Ethicon, Somerville, NJ, USA) 2/0 sutures, while a vascularized omental pedicle was fashioned and inserted into the gastric tunnel, to close the gastric defect. A vacuum-assisted drain was placed alongside the gastric repair and a Penrose drain at the duodenal repair. The band was sent for culture, which grew a multiresistant strain of* Klebsiella pneumoniae* and a sensitive strain of* Pseudomonas aeruginosa*.

Postoperatively the nasogastric tube was removed on the 4th day, and the patient was started on clear fluids, after gastrografin swallow test showed no leakage. On the 8th postoperative day she developed pneumonia, for which she received appropriate antibiotics. She was discharged on the 12th postoperative day. Gradually she regained weight (height: 165 cm, weight: 110 kgr, and BMI: 40.4) and two years later she underwent open Roux-en-Y gastric bypass.

## 3. Discussion

LAGB is generally considered a safe procedure, with less postoperative complications compared to other bariatric operations, which require more extensive dissections and anastomoses. Erosion is a relatively uncommon complication, where the band slowly erodes through the gastric wall and into the gastric lumen and becomes visible at endoscopy [5]. It is considered the most dangerous of all LAGB complications, due to its potentially life-threatening character. While the stomach is the commonest site, erosion of neighboring structures by the connecting tubing has also been reported (transverse colon, jejunum, celiac axis, and renal hilum) [7–10]. Duodenal erosion by the connecting tubing has been recently described and is a much rarer complication [11].

Most authors report erosion rates of 1–3%; however incidence varies greatly between different centers (0,23%–32%) and may reflect not only level of surgical experience and volume of patients but also length and method of followup [4, 12]. The complication presents usually late in the postoperative course, with a median time of 12–24 months from banding to erosion but can even be seen more than 10 years postoperatively [12, 13]. Erosions in the early postoperative period are usually associated with undetected intraoperative gastric wall injury [14].

The pathophysiology of erosion is still not completely understood. Early erosions are thought to be the result of microinjury to the gastric serosa, band infection, or too tight band placement [1, 5, 15, 16]. Late erosions are secondary to chronic band pressure on the stomach and local ischemia of the gastric wall [1, 5, 15, 16].

A variety of risk factors have been implicated, but no single factor can cause erosion. Although it has not been definitely proven, newer band designs (high-volume, low-pressure systems) tend to be associated less frequently with gastric erosions than older designs (low-volume, high-pressure systems), due to improved geometry and more even distribution of pressure on the gastric wall [3, 13, 17]. The surgical technique has also evolved. The perigastric approach involved more dissection around the stomach and was prone to gastric wall injuries, which could lead to erosion over time. The pars flaccida technique requires less dissection and is associated with a significantly lower erosion incidence [3, 18]. Potential risk factors may also include overfilling of the band, tension by the gastrogastric sutures, excessive vomiting, NSAIDs and other ulcerogenic drugs, smoking, alcohol, and surgeon's level of experience [3, 5, 12, 13, 15, 16]. As far as duodenal erosions are concerned, both free-floating tubing tips and attached tubing have been shown to cause erosions [11]. One may speculate that length of excess tubing could be a predisposing factor, as tubing loops may exert chronic pressure on neighboring organs.

Most cases of LAGB erosion develop gradually over time and thus are nonurgent and non-life-threatening [13]. Therefore a high index of clinical suspicion is required especially in patients with ambiguous symptomatology [19]. Almost half of the patients with erosion remain asymptomatic [3, 4, 19, 20]. Symptomatic patients present with a variety of complaints, ranging from nonspecific epigastric pain, nausea, and vomiting, as in our case, to intraabdominal abscess and generalized peritonitis [12, 15]. Unexplained weight regain and loss of satiety could be early signs of loss of restriction, due to intragastric migration of the band. Late or recurrent portsite wound infections could also point towards gastric, colonic, or duodenal erosion, with cultures typically revealing gastrointestinal, and not skin, microbial flora [11, 15]. Intraabdominal catastrophes are rare and include septic complications (abscess, peritonitis) due to free perforation or massive upper gastrointestinal hemorrhage due to erosion of adjacent vessels [9, 21].

Clinical suspicion of band erosion mandates further diagnostic workup. Contrast medium swallow test may reveal gastrografin passing from the upper to the lower gastric pouch outside the band but is usually inconclusive [15]. Abdominal CT scans can only be suggestive of erosions, showing free air or localized abscess formation around the band [15]. The cornerstone of diagnosis is upper gastrointestinal endoscopy, with retroflexed inspection of the gastric fundus [5, 12, 13, 15]. Based on endoscopic findings, erosions are classified according to Nocca as stage 1, small part of the band visible through a hole in the gastric mucosa; stage 2, partial migration (>50% of the band free in the gastric lumen); and stage 3, complete intragastric migration [6].

Management of erosions is a matter of debate. Band removal is the sine qua non of treatment, yet there is no consensus regarding timing of removal and type of future intervention. Surgical explantation can be performed as laparotomy or laparoscopy [12]. Yoon et al. advocate a 4-step procedure for repair of the gastric wall, with primary suture repair to close the defect, omental plugging into the gastric tunnel, 2 drains, and nasogastric tube for decompression [15]. Endoscopy is the least invasive modality but requires specific instruments, skilled endoscopists, and total or near-total erosion (Nocca stage 3), while it carries a relatively high risk of esophageal tearing [12]. Only asymptomatic, well-informed patients can be regularly followed, on a wait-and-watch basis, until complete erosion, thus allowing for delayed endoscopic removal [6, 20].

The majority of duodenal perforations can be effectively managed by simple repair [22]. Disruption of the suture line however is a universally feared complication and is aggravated by high intraluminal pressures and the autodigestive action of bile and pancreatic enzymes [23]. Omentopexy has comparable results, especially in perforations up to 3 cm [23]. Simultaneous gastric and duodenal erosions may contribute to higher morbidity and leakage rates.

Prognosis of gastric erosions is usually good. Evidence on LAGB-related duodenal erosions however is limited. Gastric banding causes a 360-degree sheath of reactive tissue around the band [24]. We hypothesize that this fibrosclerotic tissue may act protectively against free perforation and leakage, whereas this may not occur with duodenal erosions, which involve only the anterior wall of the first part.

In the long run, band explantation leads almost inevitably to weight regain; therefore another bariatric intervention is warranted. Selection of patients depends on local factors and the efficacy of banding on weight control. Immediate band replacement in cases without serious infection has been described but leads generally to unacceptably high reerosion rates of up to 40% [13, 20]. In cases with technical LAGB problems but good weight control, a 2-stage revision procedure, with delayed rebanding at 4–6 months, is safe and efficient and should be among the surgeon's options [13, 25–27]. On the other hand, patients with poor weight loss or noncompliance are candidates for more radical solutions and conversion to different bariatric modalities (sleeve gastrectomy, Roux-en-Y gastric bypass, and biliopancreatic diversion) [26–30].

## 4. Conclusion

Gastric erosion is an uncommon but feared complication of adjustable gastric banding for morbid obesity. Symptoms are nonspecific and diagnosis is confirmed on upper gastrointestinal endoscopy. The mainstay of treatment is surgical or endoscopic band removal, with revision or conversion to other bariatric modalities at a later stage. Erosion of the connecting tubing into the duodenum is a much rarer complication, managed adequately by primary suture repair, omentopexy, and drainage.
